# Extra-Virgin Olive Oils from Nine Italian Regions: An ^1^H NMR-Chemometric Characterization

**DOI:** 10.3390/metabo9040065

**Published:** 2019-04-03

**Authors:** Cinzia Ingallina, Antonella Cerreto, Luisa Mannina, Simone Circi, Silvia Vista, Donatella Capitani, Mattia Spano, Anatoly P. Sobolev, Federico Marini

**Affiliations:** 1 Dipartimento di Chimica e Tecnologia del Farmaco, Sapienza Università di Roma, 00185 Roma, Italy; cinzia.ingallina@uniroma1.it (C.I.); antonellacerreto@yahoo.it (A.C.); simone.circi@uniroma1.it (S.C.); vistasilvia@gmail.com (S.V.); mattia.spano@uniroma1.it (M.S.); 2 Istituto di Metodologie Chimiche, Laboratorio di Risonanza Magnetica “Annalaura Segre”, CNR, Monterotondo 00015, Roma, Italy; anatoly.sobolev@cnr.it; 3 Dipartimento di Chimica, Sapienza Università di Roma, 00185 Roma, Italy; federico.marini@uniroma1.it

**Keywords:** extra virgin olive oil (EVOO), ^1^H-NMR, linear discriminant analysis (LDA)

## Abstract

Extra-virgin olive oil (383 samples; EVOOs) of three consecutive harvesting years from nine Italian regions were collected and submitted to an ^1^H NMR-chemometric protocol to characterize the samples according to their origin (geographical area and variety). A more complete assignment of the olive oil ^1^H spectrum in CDCl_3_ and DMSO*d_6_* was reported identifying 24-methylencycolartanol. A single classification model provided the discrimination of EVOOs among the three geographical macro-areas (North, Islands, Center-South), whereas a hierarchical approach based on breaking the overall classification problem into a series of smaller linear discriminant analysis (LDA) sub-models was tested to differentiate olive oils according to their geographical regions. Specific compounds responsible for olive oil characterization were identified.

## 1. Introduction

Over recent years, newspapers and TV programs have highlighted the sensorial and nutritional properties of Italian extra-virgin olive oils (EVOOs), but they have also reported scandals concerning Italian product adulteration. Therefore, if on one hand Italian EVOOs are considered “peculiar” and “prestigious” products, on the other hand this Italian richness is constantly taken under discussion for the risk of adulteration or mislabelling. Italy represents the second largest producer of EVOOs in the world, after Spain, and Italians are also the largest consumers according to the International Olive Oil Council [1]. Due to the agronomic characteristics of the plant *Olea europaea* L., the Italian territory is especially suitable for its farming and Italy can account for 43 EVOOs provided with Protected Designation of Origin (PDO) or Protected Geographical Indication (PGI) certifications. Although EU Regulation 182/2011 [2] requires mandatory labelling reporting the geographical origin of olive oils, the actual official quality control methods (Regulation 2568/91/EEC and EU Reg. 299/2013) are unable to verify their real geographical origin [3,4]. Therefore, a potential for the mislabelling of olive oils without risk of detection exists. 

The relevance of the problem is highlighted by the fact that the European Union has launched many calls to find methods capable of checking authentication and traceability of olive oils (e.g., MEDEO project, FP6 TRACE project, FP7 Food Integrity project, Horizon 2020 call on olive oils).

The problem of geographical determination of olive oils is a difficult issue, since it also involves economic and political interests. However, the scientific world has proposed interesting possible solutions. Among the different analytical techniques, NMR spectroscopy, combined with multivariate statistical analysis, has gained an important role in geographical olive oil characterization [5]. NMR analysis provides both qualitative and quantitative information about not only the principal components of EVOOs, but also its minor compounds, including volatile aldehydes and terpenes, and healthy compounds such as squalene and β-sitosterol [6]. Furthermore, it is well known that NMR experiments are quickly performed and require neither selective extraction nor derivatization, thus reducing the experimental error and the analysis time [7]. In recent studies, the NMR analytical chemometric approach has been interfaced with the aim to build up statistical models able to characterize olive oils from Liguria [6], Apulia [8,9], Tuscany [10] and Sicily [11,12].

A combined approach, including isotope ratio mass spectrometry and NMR spectroscopy, was proposed to discriminate Italian and Tunisian olive oils [13].

In this paper, a detailed NMR-based study of olive oils from nine Italian regions over three consecutive harvesting years was carried out to investigate the presence of variables not affected by seasonal and/or climatic changes, thus identifiable as possible markers of EVOOs origin (geographical area and variety).

## 2. Results and Discussion

Italian extra-virgin olive oils (383 samples), collected from 9 Italian regions for three consecutive years, were investigated according to the NMR-chemometric protocol previously described [6,14]. It has been reported that an NMR-chemometric protocol can be a powerful tool for the geographical characterization of olive oils at different geographical scales, namely on PDO, regional, national, and Mediterranean scales. For instance, on PDO or regional scales, extra virgin olive oils from different areas of Lazio [15], Veneto [16], Tuscany [17], and Apulia [18,19] have been classified according to their geographical areas. On a national scale, a protocol based on the ^31^P NMR method has been used to characterize olive oils from different Greek areas [20,21], whereas using a ^1^H NMR methodology Italian extra virgin olive oils sampled in three harvesting years and coming from Tuscany, Lazio, and Lake Garda have been classified according to their origin [5].

Here, a study at the national scale, extended to olive oils from nine Italian regions, is reported for the first time.

### 2.1. NMR Analysis

**Figure 1:**
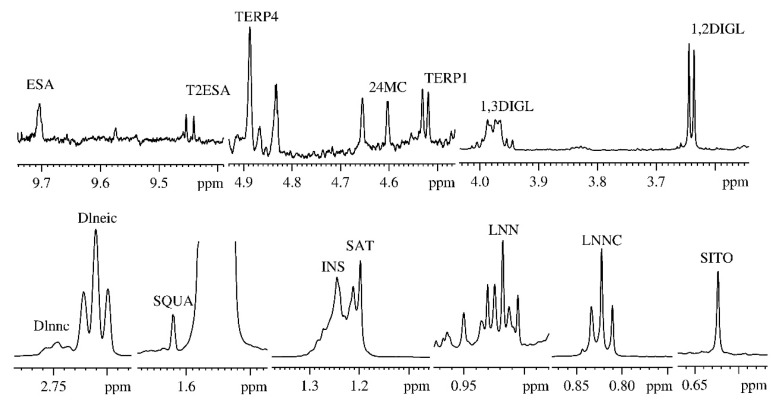
600 MHz ^1^H NMR spectrum expanded scale of an extra-virgin olive oil selected signals used in the statistical analysis, labelled as follows: SITO: methyl-18 of β-sitosterol (0.623 ppm); LNEIC: methyl of linoleic fatty acid chain (0.843 ppm); LNNC: methyl of linolenic fatty acid chain (0.910 ppm); SAT: methylenic protons of saturated fatty acid chains (1.197 ppm); SQUA: squalene (1.620 ppm); INS: methylenic protons of all unsaturated fatty acid chains (1.244 ppm); Dlneic: diallylic protons of linoleic fatty acid (2.710 ppm); Dlnnc: diallylic protons of linoleic fatty acid (2.746 ppm); 1,2DIGL: methylenic protons in α-glycerol moiety of *sn*-1,2-diglycerids (3.636 ppm); 1,3DIGL: methylenic protons in α-glycerol moiety of *sn*-1,3-diglycerids (3.988 ppm); 24MC: methylenic protons in C31 of 24-methylenecycloartenol (4.609 ppm); TERP1: terpene 1 (4.541 ppm); TERP4: terpene 4 (4.885 ppm); T2ESA: *trans*-2-hexanal (9.454 ppm); ESA: hexanal (9.704 ppm).

According to the NMR protocol reported in literature [6], the intensity of 15 ^1^H NMR signals was measured and submitted to the chemometric analysis (see Figure 1 and Table 1).

**Figure 2:**
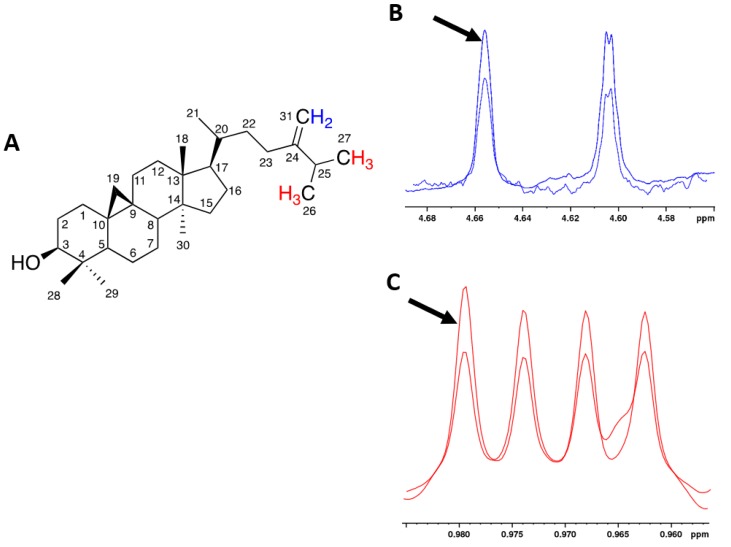
(**A**) Chemical structure of 24MC. (**B**) CH_2_-31 ^1^H signals in the ^1^H NMR olive oil spectrum. The arrow indicates the same signals after the 24MC standard addition. (**C**) CH_3_-26 and CH_3_-27 ^1^H signals in the ^1^H NMR olive oil spectrum. The arrow indicates the same signals after the 24MC standard addition.

A more complete assignment of the olive oil ^1^H spectrum was obtained by means of the addition of 24-methylenecycloartanol (24MC) synthesized standard [22] to the olive oil sample and 2D experiments. The ^1^H and ^13^C assignment of the 24MC, obtained by 2D experiments (see Table S1), agreed with the assignment reported previously [23]. Signals at 4.604 and 4.665 ppm, previously reported generically as terpenes [13,24], were assigned to CH_2_-31 methylene protons of 24MC, whereas the two doublets at 0.968 and 0.974 previously reported as wax [13,24], were assigned to CH_3_-26 and CH_3_-27 protons, respectively [25] (see Figure 2).

### 2.2. Global Model

The NMR data (signal intensity) concerning olive oil samples collected during the three harvesting years were gathered in a matrix (dimensions: 383 × 15) for the chemometric data processing. In particular, since the aim of the study was, in the first place, to discriminate olive oils by the different Italian regions, classification models had to be built using linear discriminant analysis (LDA) [26]. In this context, in order to be able to validate the results [27] of predictive modeling on an independent (external) set of samples, prior to any model building the total set of samples was split into a training and a test set. To do so and guarantee that all classes were appropriately represented in both sets, the Kennard–Stone algorithm [28] was applied to each category individually with a 70:30 splitting ratio. Accordingly, 269 samples (36 from Calabria, 47 from Lazio, 15 from Liguria, 16 from Lombardy, 27 from Molise, 53 from Apulia, 24 from Sardinia, 31 from Sicily and 20 from Tuscany) were included in the training set, and the remaining 114 (15 from Calabria, 20 from Lazio, 6 from Liguria, 7 from Lombardy, 11 from Molise, 23 from Apulia, 10 from Sardinia, 13 from Sicily and 9 from Tuscany) were left aside as the external test set. 

At first, the possibility of building a single classification model to discriminate among all the investigated regions was attempted. To do so, LDA was applied to the training set (since the classification technique naturally embeds autoscaling, no further data preprocessing was needed) in order to define the decision surfaces separating the classes in the multivariate hyperspace of the variables, and the results obtained on the same set of samples during the model building (calibration) stage are summarized in Table 2. By looking at Table 2, it is evident how, when trying to use a single model to discriminate olive oils among all the investigated regions, some categories (in particular, the Northern Regions and the Islands) are classified better than the others. This observation is confirmed when the calculated LDA model is applied to the external validation set (Table 2), although the performances on the test set are slightly worse than those obtained in calibration. The corresponding confusion matrices, reported in Tables S2 and S3 for the calibration and the validation set, respectively, show in detail how the classification/prediction errors are distributed.

Altogether, these results are not completely unexpected. In fact, the extensive sampling results in different sources of variability affecting the investigated samples. Geographical areas with different pedoclimatic conditions, specific cultivars, and harvesting year may have a relevant impact on the variance observed among the metabolite profiles. However, using quadratic instead of linear discriminant analysis did not significantly improve results. 

Accordingly, in a second stage of chemometric processing to deal with the observed complexity, a hierarchical approach based on breaking the overall classification problem into a series of smaller sub-models to be sequentially applied was tested. 

**Figure 3:**
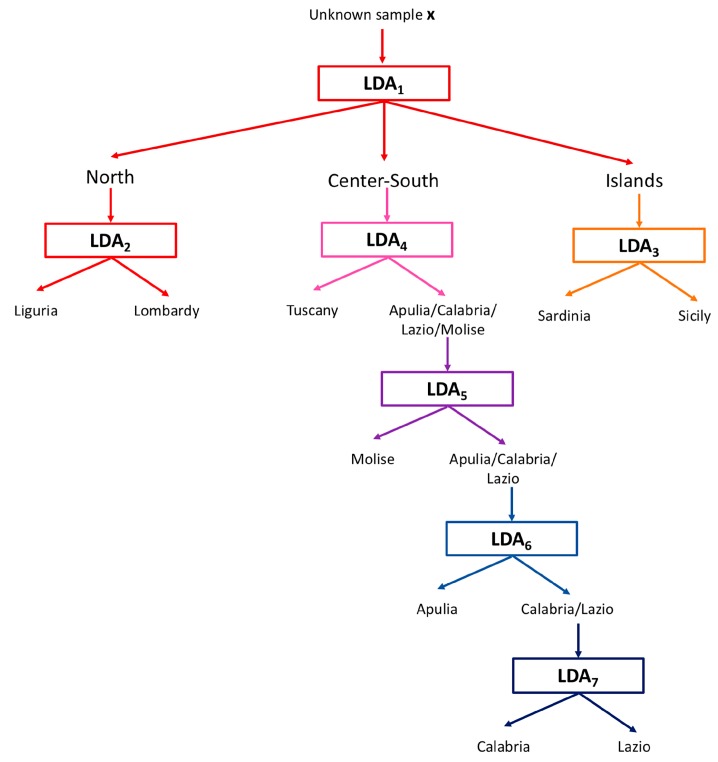
Scheme of the hierarchical classification approach based on the calculation and application of successive LDA models.

In detail, at first a classification model to discriminate among macro-geographical areas, that is, Northern (Liguria and Lombardy), Central-Southern (Tuscany, Lazio, Molise, Apulia and Calabria) and Island (Sicily and Sardinia) regions, was built using LDA. Successively, additional models discriminating among the individual members of these macro-geographical areas were built: One LDA model was built to discriminate samples from Liguria and Lombardy, another one to discriminate oils from Sicily from those of Sardinia and, lastly, four successive models were built to differentiate all the Central-Southern regions (at first Tuscany from the other four, i.e., Calabria, Lazio, Molise and Apulia; then, Molise from the remaining three, Apulia from the remaining two and, finally, Calabria from Lazio). The scheme of this hierarchical approach is illustrated in Figure 3. In all these cases, the model parameters (i.e., the parameters governing the decision boundaries separating the categories) were calculated on the basis of the training samples and, successively, validation was carried out by applying the model on the external test samples. The results of both the calibration and the validation phases are summarized in Table 3.

**Figure 4:**
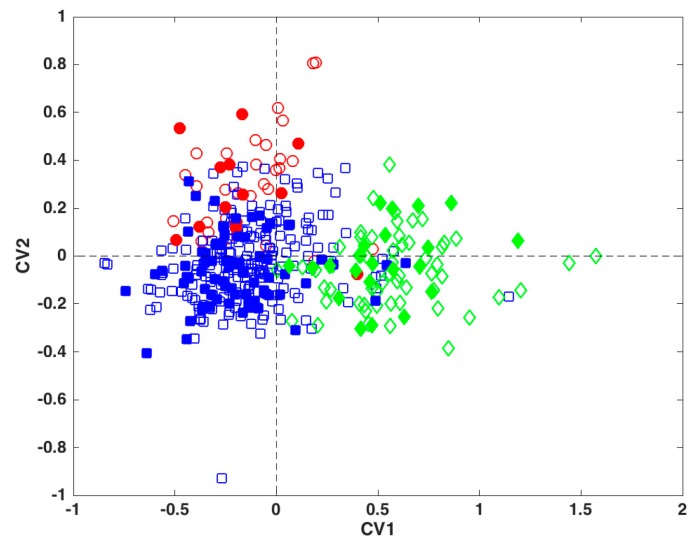
Projection of the training (empty symbols) and test samples (filled symbols) onto the space spanned by the two canonical variates of the LDA model. Legend: Red circles—North; blue squares—Center-South; green diamonds—Islands.

The LDA model built to discriminate among the three geographical macro-areas provides very good classification ability not only on the training data, but also in validation, where the correct classification rate is always above 84% and higher than 90% for the category Islands. The separation among the classes can also be visually appreciated by looking at the projection of the training and test samples onto the space spanned by the two canonical variates (Figure 4), where it is evident how a different metabolic profile characterizes the Northern Italy and the Island samples with respect to the rest of the investigated regions. 

In particular, by looking at the canonical weights (see supplementary Table S4), it is possible to affirm that the Northern Italy samples are characterized by higher values of 24MC and TERP1 and lower values of SAT and LNEIC, whereas the Island olive oils have higher amounts of TERP4, Dlneic, SQUA and LNEIC, and lower concentrations of TERP1 and SITO. Lastly, Central-Southern Italian samples are characterized by higher values of SAT, LNNC and SITO, and lower values of TERP4 and 24MC.

Having shown that it is possible to discriminate EVOOs from the North, Center-South and Islands, the successive step was to build models in order to differentiate the regions within each of the three macro-areas. When considering the Northern Italian samples, a model was then built to separate Liguria from Lombardy, keeping, for the samples belonging to these two categories, the same training/test split as in the complete data set. Accordingly, 31 samples (15 from Liguria, 16 from Lombardy) constituted the training set and 13 olive oils (6 from Liguria, 7 from Lombardy) the external validation set. The LDA model was built on the training samples and validated on the test set and the results are reported in Table 3. The results show how an almost perfect classification can be accomplished (only one sample misclassified in the calibration stage) when trying to discriminate Ligurian olive oils from samples harvested in Lombardy. An inspection of the model parameters (coefficients of the decision surface and weights of the only canonical variate which could be extracted from the data, reported in supplementary material as Table S5, together with the corresponding canonical score plot in supplementary Figure S1) suggests that most of the metabolites are higher in samples coming from Liguria, the only exceptions being 1,2-DIGL, SQUA, SAT and LNNC.

Analogously, another model was built on the samples from the Islands, in order to discriminate between those harvested in Sicily and Sardinia. In this case, 55 samples (24 from Sardinia, 31 from Sicily) constituted the training set and 23 olive oils (10 from Sardinia, 13 from Sicily) were left out as the external test set. The LDA model was then built on the training samples and validated on the test ones; the results are reported in Table 3. It can be observed how the correct classification rate is higher than 90% both in calibration and in validation; in particular, during the validation phase, only one sample from Sicily was wrongly predicted, suggesting that the model has a high accuracy in discriminating the olive oils coming from the two main Italian islands. Inspection of the model parameters for the sake of chemical interpretation of the classification results (projection of the samples onto the only canonical variate is displayed as supplementary Figure S2, while the corresponding canonical weights are reported in supplementary Table S6) highlights how Sardinian samples are characterized by higher concentrations of 24MC, Dlneic, SAT and LNEIC, whereas olive oils from Sicily are richer in TERP4, TERP1, 1,3DIGL and 1,2DIGL.

Following this, attention was focused on the samples from central and southern Italy which, due to their closer similarity, were difficult to discriminate. In detail, at first it was decided to build a model for differentiating Tuscan olive oils from the others (i.e., Calabria, Lazio, Molise and Apulia). As shown in Table 4, the corresponding LDA model was able to correctly classify more than 85% of the samples both in calibration and in validation, with the predictive ability on the test set being around 90%. 

As far as the interpretation of the observed results is concerned, inspection of the model parameters (projection of the samples onto the only canonical variate is displayed as supplementary Figure S3, while the corresponding canonical weights are reported in supplementary Table S7) evidenced how olive oils from Tuscany, with respect to the other samples from central and southern Italian regions, are characterized by a higher content of TERP4, 24MC, TERP1, 1,2DIGL and SITO, and a lower content of T2ESE, 1,3DIGL and SQUA.

Then, another model was built to differentiate samples from Molise and the remaining Center-South regions (Lazio, Apulia and Calabria). The results of the training and test sets are reported in Table 4. 

In this case, the correct classification rate was higher than 80% both in calibration and in validation, as shown in Table 4. When trying to interpret the observed results in chemical terms by inspecting the model parameters (projection of the samples onto the only canonical variate is displayed as supplementary Figure S4, while the corresponding canonical weights are reported in supplementary Table S8), one could infer that olive oils from Molise are characterized by a higher content of T2ESE, 24MC and SQUA, and a lower content of TERP4 and 1,2DIGL.

Apulia olive oils were then discriminated from Calabria and Lazio ones by means of another LDA model, the results of which are reported in Table 4 and indicate a predictive ability of around 80% for both categories. An inspection of the model parameters (projection of the samples onto the only canonical variate is displayed as supplementary Figure S5, while the corresponding canonical weights are reported in supplementary Table S9) suggests that olive oils from Apulia have a higher content of TERP4 and 1,2DIGL with respect to Lazio and Calabria samples, and a lower amount of SQUA and SITO.

Lastly, a final model was built to discriminate samples from Lazio with respect to those from Calabria. Compared with the other models, this last model showed a lower classification accuracy, as shown in Table 4. Nevertheless, it was still able to correctly predict about 87% of the validation samples from Calabria. 

Interpretation of the classification model in chemical terms (projection of the samples onto the only canonical variate is displayed as supplementary Figure S6, while the corresponding canonical weights are reported in supplementary Table S10) indicates that most of the metabolites are higher in the olive oils from Calabria (in particular, TERP1, SQUA), with the only relevant exceptions being T2ESE, Dlneic, and LNEIC, which are higher in Lazio samples.

The results obtained when applying the whole hierarchical approach to the data set are summarized in Table 5. Comparing the results in Table 5 with those of the single global model reported in Table 2, it is evident that the use of a hierarchical approach leads to an improvement of the classification ability for almost all the classes, and in the few cases where results are not better, they remain at least comparable.

The use of a hierarchical approach appears to have various advantages over a single global model. First of all, it leads to a higher classification accuracy for most of the classes. Moreover, the individual sub-models are easier and more straightforward to interpret from a chemical standpoint. Additionally, if one wants to focus on a discrimination among specific regions or groups of regions, the sub-models can be used independently outside of the hierarchical classification pipeline.

## 3. Materials and Methods 

### 3.1. Sampling 

EVOO samples, provided by different farms selected by the UNAPROL Italian consortium of olive oil producers, were collected for three consecutive harvesting years from different Italian regions. All olive oil samples were provided every year by the same local producers associated to UNAPROL to assure the same geographical provenience. In particular, 151 samples were collected from 8 regions in 2009/10 (Lombardia, *n* = 2; Tuscany, *n* = 10; Lazio, *n* = 33; Molise, *n* = 18; Apulia, *n* = 32, Calabria, *n* = 25; Sicily, *n* = 24; Sardinia, *n* = 7), 122 samples from 9 regions in 2010/11 (Lombardia, *n* = 6; Liguria, *n* = 10; Tuscany, *n* = 9; Lazio, *n* = 10; Molise, *n* = 10; Apulia, *n* = 27; Calabria, *n* = 25; Sicily, *n* = 8; Sardinia, *n* = 13) and 114 samples in 8 regions in 2011/12 (Lombardia, *n* = 15; Liguria, *n* = 11, Tuscany, *n* = 15; Lazio, *n* = 24; Molise, *n* = 10; Apulia, *n* = 13, Sicily, *n* = 12; Sardinia, *n* = 14). 

In order to prevent any alteration of the labile product, samples were shelved protected from light, oxygen and heat in proper containers [29,30,31]. Temperature was included in the range 10–15 °C, to prevent the olive oil solidification and the consequent decrease of the phenols concentration at low temperature, and the triggering of the oxidative reactions at higher temperature.

### 3.2. NMR Analyses 

Olive oil samples (20 μL) were dissolved in 700 μL of deuterated chloroform (CDCl_3_) and 20 μL of deuterated dimethylsulfoxide (DMSO*d_6_*) was added to increase the sample stability. To avoid degradation, the ^1^H spectra are acquired within an hour from the sample preparation. 

The ^1^H-NMR spectra were performed on a Bruker AVANCE 600 spectrometer operating at the protonic frequency of 600.13 MHz (14.3 T), equipped with a 5 mm probe for the protons. The signals of residual non-deuterated solvents (CHCl_3_ and DMSO) at 7.260 ppm and at 2.526 ppm, respectively, were used to verify the spectrum resolution. 

The acquisition parameters used were: Time domain (TD)= 64 K; spectral window (SW) = 18.5 ppm; O1 = 4580 Hz; receiver gain (RG) = 16; 90° degrees pulse optimized for each spectrum; delay (D1) = 0.5 s; acquisition time (AQ) = 2.96 s; number of scansion (NS) = 1024; dummy scans (DS) = 4; T = 300 K.

The ^1^H spectra were processed using the program TOPSPIN (version 1.3) with the following parameters: LB= 0.3 Hz (exponential function); SI = 64 K (zero filling); manual correction of the phase; spectrum normalization with respect to the signal due to the methylene protons in β to the carbonyl (2.251 ppm) whose intensity is set equal to 1000; semi-manual correction of the base line (cubic spline baseline correction); and automatic reading of the intensity.

A 24-methylenecycloartanol standard was synthesized from the commercially available oryzanol (Merck CDS021604-1G containing 40% of 3-O-ferulylcycloartenol and 40% of 3-O-ferulyl-24-methylenecycloartanol) following the reported procedure [22].

### 3.3. Chemometrics 

In order to differentiate the olive oil samples from the different regions under investigation, a classification strategy based on linear discriminant analysis was adopted. Classification methods aim at building predictive models for qualitative (discrete-valued) responses; each of the possible values of the responses is deemed a category (or class). In this context, classification methods formulate models to predict, for each sample, the value of this qualitative response, that is, its class based on the experimental measurements collected. This is accomplished in a probabilistic way, that is, by evaluating for each individual the probabilities of the different possible responses (i.e., of the sample being class 1, class 2, …, up to class K) and then selecting the most probable outcome. Among the different classification techniques, linear discriminant analysis (LDA) assumes that within each category, data are normally distributed, so that calculation of the probability that a sample is class k only requires the estimation of the centroid and covariance matrix of such distribution: for this reason, methods such as LDA are called parametric. Accordingly, the probability that a sample is from (or, as it often said, belongs to) class k, $p_{k}\left(x\right)$, can be calculated as

(1) $$p_{k}\left(x\right)=\frac{C_{k}}{2\pi^{\frac{d}{2}}\left|S\right|}e^{-\frac{1}{2}{\left(x-{\bar{x}}_{k}\right)}^{T}S^{-1}\left(x-{\bar{x}}_{k}\right)}$$

where $x-{\bar{x}}_{k}$ is the class centroid and S is the pooled within-class variance/covariance matrix, which is assumed to be the same for each category, whereas $C_{k}$ is a normalization constant.

During the model building stage, the training samples from the different categories are used to estimate the parameters $x-{\bar{x}}_{k}$, S, $C_{k}$ in Equation (1). Once the parameters of the probability functions for the different classes are estimated, predictions on an unknown sample are accomplished by inserting the measured profile x in Equation (1) for the different categories in order to calculate the probabilities of it belonging to each of the investigated classes. The sample is then predicted to belong to the category corresponding to the highest value of the probability.

In this study, at first a global model considering all the categories involved was calculated. In a second stage, a hierarchical classification approach was adopted, in which LDA was repeatedly applied to discriminate among groups of decreasing heterogeneity.

## 4. Conclusions

A deep characterization of the Italian extra-virgin olive oils was performed by means of a combination of NMR spectroscopy and chemometric analysis. A single classification model provided the discrimination of Italian EVOOs among the North, Islands, and Center-South geographical areas. Northern Italy samples were characterized by higher values of 24MC and TERP1 and lower values of SAT and LNEIC, whereas the Island olive oils had higher amounts of TERP4, Dlneic, SQUA and LNEIC, and lower concentrations of TERP1 and SITO. Central-Southern Italian samples showed higher values of SAT, LNNC and SITO and lower values of TERP4 and 24MC.

A hierarchical approach, based on breaking the overall classification problem down into a series of smaller LDA sub-models, was tested in order to differentiate the regions within each of the three identified macro-areas. A good classification accuracy for most of the classes corresponding to Italian regions was obtained together with the model interpretation based on chemical composition.

## Figures and Tables

**Table 1:** Signals in the ^1^H-NMR olive oil spectrum used for the statistical analysis.

Assignment	NMR Resonances (ppm)
Methyl 18 of β-sitosterol (SITO)	0.623
Linoleic acid methyl group (LNEIC)	0.843
Linolenic acid methyl group (LNNC)	0.910
Methylenic protons of the saturated fatty acids chains (SAT)	1.197
Methylenic protons of the unsaturated fatty acids chains (INS)	1.244
Squalene (SQUA)	1.620
Diallylic protons of the linoleic acid (Dlneic)	2.710
Diallylic protons of the linolenic acid (Dlnnc)	2.746
*Sn*-1,2-diglycerids α methylenic protons (1,2DIGL)	3.636
*Sn*-1,3-diglycerids α methylenic protons (1,3DIGL)	3.988
Terpene 1 (TERP1)	4.541
24-methylenecycloartanol (24MC)	4.609
Terpene 4 (TERP4)	4.885
*Trans*-2-Hexanal (T2ESA)	9.454
Hexanal (ESA)	9.704

**Table 2:** Results of linear discriminant analysis (LDA) classification using a global model discriminating all nine Italian regions: Correct classification rates on the training (calibration) and the test (validation) sets.

Region	Calibration	Validation
Calabria	36.11 (13/36) ^a^	40.00 (6/15)
Lazio	44.68 (21/47)	60.00 (12/20)
Liguria	80.00 (12/15)	66.67 (4/6)
Lombardy	75.00 (12/16)	100.00 (7/7)
Molise	81.48 (22/27)	63.64 (7/11)
Apulia	49.06 (26/53)	43.48 (10/23)
Sardinia	91.67 (22/24)	90.00 (9/10)
Sicily	74.19 (23/31)	61.54 (8/13)
Tuscany	60.00 (12/20)	66.67 (6/9)

^a^ The numbers in parentheses indicate the number of correctly classified samples and the total number of samples for a particular class, respectively.

**Table 3:** LDA results for the classification according to macro-geographical areas (LDA_1_), and for the discrimination between Northern Italy regions (LDA_2_) or Island regions (LDA_3_): Correct classification rates on the training (calibration) and the test (validation) sets.

Region	Calibration	Validation
Macro-geographical area (LDA_1_)		
North	80.65 (25/31) ^a^	84.62 (11/13)
Center-South	77.05 (141/183)	85.90 (67/78)
Islands	92.73 (51/55)	91.30 (21/23)
Northern Italian regions (LDA_2_)		
Liguria	93.33 (14/15)	100.00 (6/6)
Lombardy	100.00 (16/16)	100.00 (7/7)
Island regions (LDA_3_)		
Sardinia	95.83 (23/24)	100.00 (10/10)
Sicily	90.32 (28/31)	92.31 (12/13)

^a^ The numbers in parentheses indicate the number of correctly classified samples and the total number of samples for a particular class, respectively.

**Table 4:** Results of LDA for the hierarchical discrimination among Tuscany, Molise, Apulia and Calabria olive oils with respect to samples from other Center-South regions: Correct classification rates on the training (calibration) and the test (validation) sets.

Region	Calibration	Validation
Tuscany	85.00 (17/20) ^a^	88.89 (8/9)
Others (Calabria, Lazio, Molise, Apulia)	88.96 (145/163)	91.30 (63/69)
Molise	85.19 (23/27)	81.82 (9/11)
Others (Lazio, Apulia, Calabria)	84.56 (115/136)	84.48 (49/58)
Apulia	81.13 (43/53)	82.61 (19/23)
Others (Lazio, Calabria)	79.52 (66/83)	80.00 (28/35)
Calabria	69.44 (25/36)	86.67 (13/15)
Lazio	70.22 (33/47)	60.00 (12/20)

^a^ The numbers in parentheses indicate the number of correctly classified samples and the total number of samples for a particular class, respectively.

**Table 5:** Results of the overall LDA hierarchical classification of the olive oil samples with respect to the region of origin: Correct classification rates on the training (calibration) and the test (validation) sets.

Region	Calibration	Validation
Calabria	73.33 (24/36) ^a^	73.33 (11/15)
Lazio	63.83 (30/47)	60.00 (12/20)
Liguria	86.67 (13/15)	83.33 (5/6)
Lombardy	81.25 (13/16)	100.00 (7/7)
Molise	85.19 (23/27)	72.73 (8/11)
Apulia	66.04 (35/53)	65.22 (15/23)
Sardinia	95.83 (23/24)	100.00 (10/10)
Sicily	77.42 (24/31)	76.92 (9/13)
Tuscany	65.00 (13/20)	66.67 (6/9)

^a^ The numbers in parentheses indicate the number of correctly classified samples and the total number of samples for a particular class, respectively.

## Supplementary Materials

The following are available online at https://www.mdpi.com/2218-1989/9/4/65/s1, Figure S1: LDA model discriminating oil samples from Northern Italy (LDA2), Figure S2: LDA model discriminating oil samples from Italian Islands (LDA3), Figure S3: LDA model discriminating oil samples from Tuscany from other Central/Southern Italy (Apulia, Calabria, Lazio and Molise) samples (LDA4), Figure S4: LDA model discriminating oil samples from Molise from other Central/Southern Italy (Apulia, Calabria and Lazio) samples (LDA5), Figure S5: LDA model discriminating oil samples from Apulia from other Central/Southern Italy (Calabria and Lazio) samples (LDA6), Figure S6: LDA model discriminating between oil samples from Calabria and Lazio (LDA7), Table S1: ^1^H and ^13^C NMR data of 24MC synthesized standard in CDCl_3_, Table S2: Global LDA model discriminating samples from all 9 Italian regions. Confusion matrix for the training samples (calibration results), Table S3: Global LDA model discriminating samples from all 9 Italian regions. Confusion matrix for the test samples (validation results), Table S4: LDA model discriminating oil samples according to Italian macro-greographical areas (LDA1): Variable weights on the two canonical variates., Table S5: LDA model discriminating oil samples from Northern Italy (LDA2), Table S6: LDA model discriminating oil samples from Italian Islands (LDA3), Table S7: LDA hierarchical model discriminating oil samples from Tuscany from other Central/Southern Italy (Apulia, Calabria, Lazio and Molise) samples (LDA4), Table S8: LDA hierarchical model discriminating oil samples from Molise from other Central/Southern Italy (Apulia, Calabria and Lazio) samples (LDA5), Table S9: LDA hierarchical model discriminating oil samples from Apulia from other Central/Southern Italy (Calabria and Lazio) samples (LDA6), Table S9: LDA hierarchical model discriminating oil samples from Apulia from other Central/Southern Italy (Calabria and Lazio) samples (LDA6), Table S10: LDA hierarchical model discriminating between oil samples from Calabria and Lazio (LDA7).
