# Supplementary material for: Extra-Virgin Olive Oils from Nine Italian Regions: An 1H NMR-Chemometric Characterization
Source: Metabolites. 2019 Apr 3;9(4):65. doi: 10.3390/metabo9040065 (PMC6523492; doi:10.3390/metabo9040065)
Supplement: Supplementary file 1 [file metabolites-09-00065-s001.pdf]

Supplementary material:
Extra-virgin olive oils from nine Italian regions: an ^{1}H NMR-chemometric characterization

Cinzia Ingallina [1], Antonella Cerreto [1], Luisa Mannina [1,2,*], Simone Circi [1], Silvia Vista[1], Donatella Capitani [2*], Mattia Spano[1] , Anatoly P. Sobolev [2] and Federico Marini [3]

1 *Dipartimento di Chimica e Tecnologia del Farmaco, Sapienza Università di Roma*; cinzia.ingallina@uniroma1.it; antonellacerreto@yahoo.it; luisa.mannina@uniroma1.it; simone.circi@uniroma1.it; vistasilvia@gmail.com
2 *Istituto di Metodologie Chimiche, Laboratorio di Risonanza Magnetica "Annalaura Segre", CNR, Monterotondo, Roma*; donatella.capitani@cnr.it; anatoly.sobolev@cnr.it
3 *Dipartimento di Chimica, Sapienza Università di Roma*; federico.marini@uniroma1.it
* Correspondence: luisa.mannina@uniroma1.it; Tel.: +39-06-4991-3735; donatella.capitani@cnr.it; Tel.: +39-06-9067-2700

Table S1. ^{1}H and ^{13}C NMR data of 24MC synthesized standard in $CDCl_3$

Group	1H (ppm)	Multiplicity [J(Hz)]	13C (ppm)
CH_2-1	1.25	m	31.9
CH_2-1	1.56	m	31.9
CH_2-2	1.76	m	30.4
CH_2-2	1.57	m	30.4
CH_2-3	3.28	m	78.8
CH-5	1.30	m	47.2
CH_2-6	0.80	m	21.1
CH_2-6	1.59	m	21.1
CH_2-7	1.08	m	26.0
CH_2-7	1.33	m	26.0
CH-8	1.51	m	48.0
CH_2-11	1.12	m	26.5
CH_2-11	2.00	m	26.5
CH_2-12	1.62	m	33.0
CH_2-15	1.31	m	35.7
CH_2-16	1.92	m	28.2
CH_2-16	1.30	m	28.2
CH-17	1.61	m	52.4
CH_3-18	1.02	s	18.1
CH_2-19 *eso*	0.33	d [4]	29.8
CH_2-19 *endo*	0.56	d [4]	29.8

CH-20	1.43	m	36.6
CH_3-21	0.90	d [5]	18.4
CH_2-22	1.16	m	35.0
CH_2-22	1.57	m	35.0
CH_2-23	1.89	m	31.4
CH_2-23	2.13	ddd [14.6, 11.3, 4.9]	31.4
CH-25	2.24	sept [6.8]	33.8
CH_3-26/27	1.03	d [6.8]	21.9
CH_3-26/27	1.04	d [6.8]	22.0
CH_3-28	0.97	s	25.7
CH_3-29	0.83	s	14.2
CH_3-30	0.91	s	19.5
CH_2-31	4.66	d [1.4]	105.9
CH_2-31	4.72	s	105.9

m = multiplet
s = singlet
d = doublet
ddd = double double doublet
sept = septuplet

Reference

Benabdelaziz, I.; Haba, H.; Lavaud, C.; Benkhaled, M. Triterpenoids and Flavonoid from *Scorzonera undulata* ssp. alexandrina. *Int J Chem Biol Sci* **2014**, *5*, 1-5.

Table S2: Global LDA model discriminating samples from all 9 Italian regions. Confusion matrix for the training samples (calibration results)

		Predicted class								
		**Calabria**	**Lazio**	**Liguria**	**Lombardy**	**Molise**	**Apulia**	**Sardinia**	**Sicily**	**Tuscany**
**True class**	**Calabria**	13	6	0	6	2	6	3	0	0
	**Lazio**	6	21	0	7	3	6	1	2	1
	**Liguria**	0	0	12	0	0	1	0	0	2
	**Lombardy**	0	0	0	12	0	1	0	1	2
	**Molise**	1	0	1	2	22	0	1	0	0
	**Apulia**	7	6	0	5	0	26	0	5	4
	**Sardinia**	0	0	0	0	0	1	22	1	0
	**Sicily**	1	0	0	0	0	2	5	23	0
	**Tuscany**	0	1	1	2	0	4	0	0	12

Table S3: Global LDA model discriminating samples from all 9 Italian regions. Confusion matrix for the test samples (validation results)

		Predicted class								
		**Calabria**	**Lazio**	**Liguria**	**Lombardy**	**Molise**	**Apulia**	**Sardinia**	**Sicily**	**Tuscany**
**True class**	**Calabria**	6	4	0	0	0	3	1	1	0
	**Lazio**	4	12	0	1	1	1	0	0	1
	**Liguria**	0	0	4	0	0	0	1	0	1
	**Lombardy**	0	0	0	7	0	0	0	0	0
	**Molise**	0	1	0	3	7	0	0	0	0
	**Apulia**	4	2	0	1	2	10	0	2	2
	**Sardinia**	0	0	0	0	0	0	9	1	0
	**Sicily**	1	0	0	0	1	0	3	8	0
	**Tuscany**	0	1	0	2	0	0	0	0	6

Table S4: LDA model discriminating oil samples according to Italian macro-greographical areas (LDA_1): Variable weights on the two canonical variates.

Variable	CV1	CV2
ESA	0.0106	0.0803
T2ESE	-0.0224	0.0124
TERP4	0.2214	0.4783
TERP2	-0.2497	0.8732
TERP1	-0.8551	0.1492
1,3DIGL	0.1216	0.0611
1,2DIGL	0.1285	0.1035
Dlnnc	-0.1126	0.1695
Dlneic	0.5476	0.0106
SQUA	0.1758	0.4507
INS	-0.2021	0.2404
SAT	0.0306	0.4499
LNNC	-0.0318	0.2831
LNEIC	0.5441	0.0926
SITO	-0.2249	0.2697

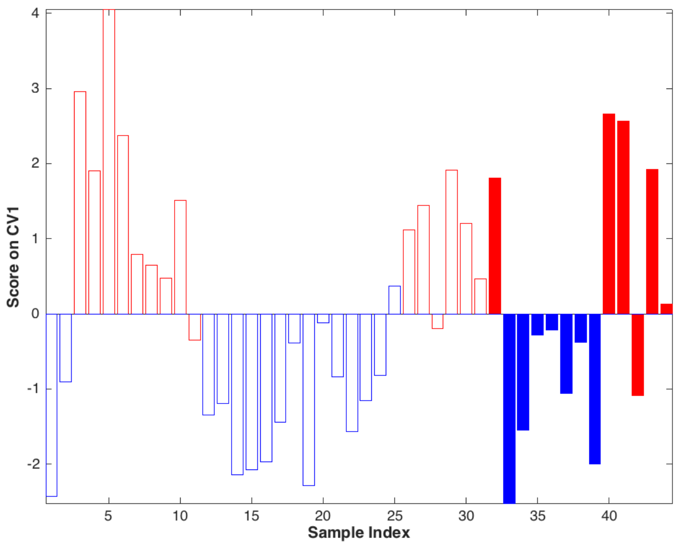

Figure S1. LDA model discriminating oil samples from Northern Italy (LDA_2): Projection of the training (empty bars) and test samples (filled bars) onto the space spanned by the only Canonical Variates of the LDA model. Legend: Red bars – Liguria; Blue bars – Lombardia.

TableS5: LDA model discriminating oil samples from Northern Italy (LDA_2): Variable weights on the canonical variate.

Variable	CV1
ESA	0.3881
T2ESE	0.3857
TERP4	0.0177
TERP2	0.3358
TERP1	0.0788
1,3DIGL	0.1371
1,2DIGL	0.1282
Dlnnc	0.1461
Dlneic	0.0084
SQUA	-0.3472
INS	0.1289
SAT	-0.1473
LNNC	-0.1451
LNEIC	-0.0059
SITO	0.2892

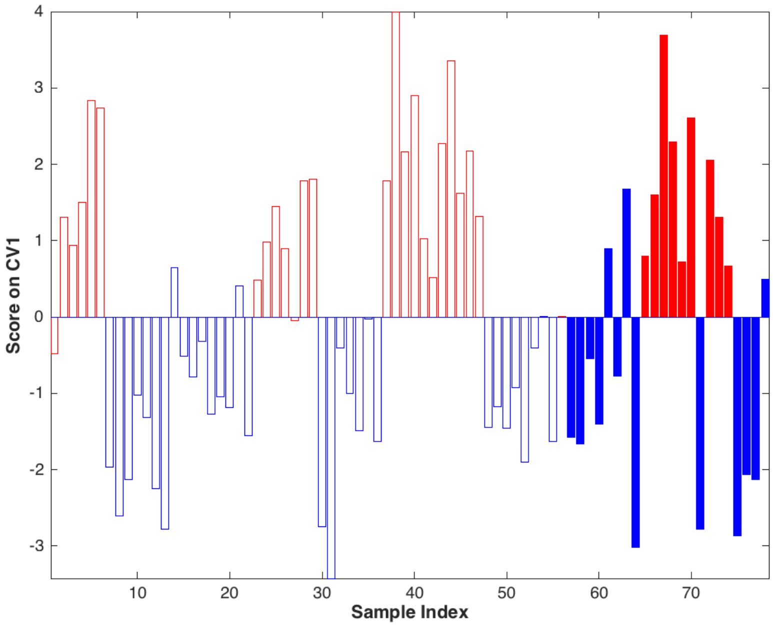

Figure S2. LDA model discriminating oil samples from Italian Islands (LDA_3): Projection of the training (empty bars) and test samples (filled bars) onto the space spanned by the only Canonical Variates of the LDA model. Legend: Red bars – Sardinia; Blue bars – Sicily.

Table S6: LDA model discriminating oil samples from Italian Islands (LDA_3): Variable weights on the canonical variate.

Variable	CV1
ESA	0.1130
T2ESE	0.3547
TERP4	-0.4577
TERP2	0.8208
TERP1	-0.1341
1,3DIGL	-0.5332
1,2DIGL	-0.3891
Dlnnc	0.0719
Dlneic	0.4333
SQUA	0.3653
INS	-0.0646
SAT	0.4730
LNNC	-0.2206
LNEIC	0.3078
SITO	-0.1042

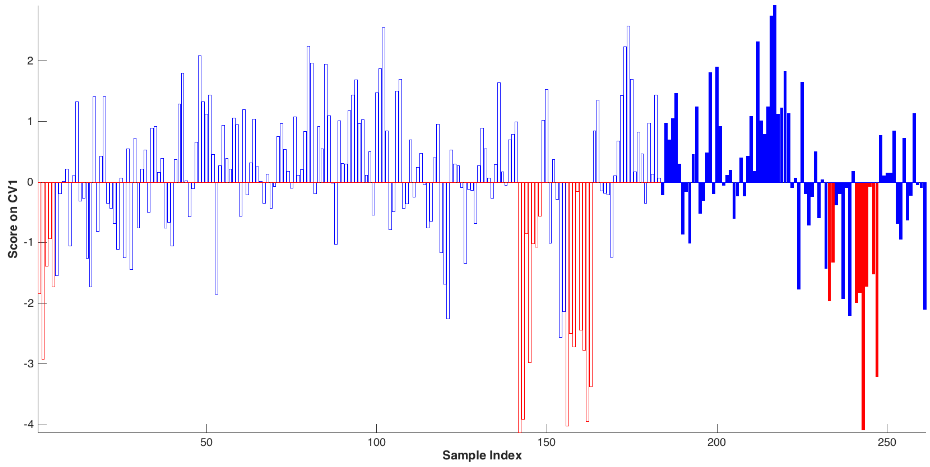

Figure S3. LDA model discriminating oil samples from Tuscany from other Central/Southern Italy (Apulia, Calabria, Lazio and Molise) samples (LDA_4): Projection of the training (empty bars) and test samples (filled bars) onto the space spanned by the only Canonical Variates of the LDA model. Legend: Red bars – Tuscany; Blue bars – other Central/Southern Italy (Apulia, Calabria, Lazio and Molise).

Table S7: LDA hierarchical model discriminating oil samples from Tuscany from other Central/Southern Italy (Apulia, Calabria, Lazio and Molise) samples (LDA_4): Variable weights on the canonical variate.

Variable	CV1
ESA	0.0554
T2ESE	0.2195
TERP4	-0.2993
TERP2	-0.7505
TERP1	-0.2278
1,3DIGL	0.3761
1,2DIGL	-0.3643
Dlnnc	0.1108
Dlneic	0.1481
SQUA	0.5108
INS	-0.0552
SAT	0.0868
LNNC	-0.0556
LNEIC	0.1372
SITO	-0.3445

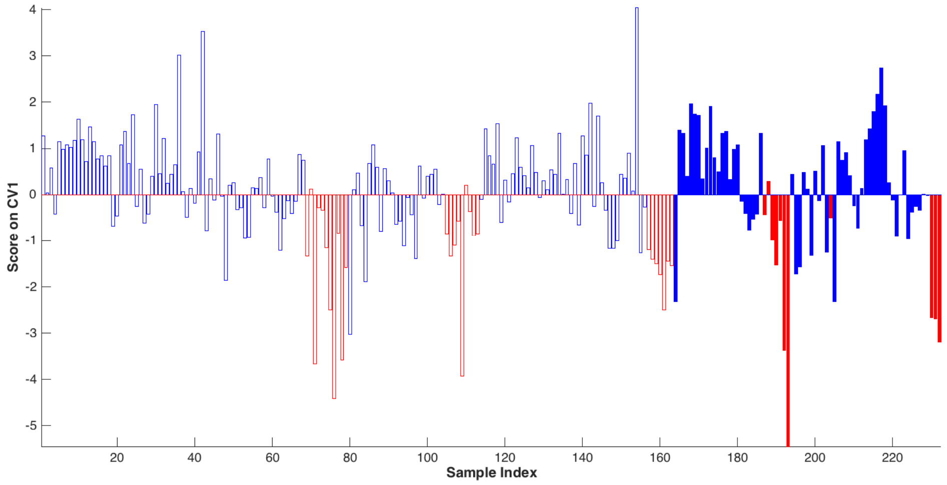

Figure S4. LDA model discriminating oil samples from Molise from other Central/Southern Italy (Apulia, Calabria and Lazio) samples (LDA_5): Projection of the training (empty bars) and test samples (filled bars) onto the space spanned by the only Canonical Variates of the LDA model. Legend: Red bars – Molise; Blue bars – other Central/Southern Italy (Apulia, Calabria and Lazio).

Table S8: LDA hierarchical model discriminating oil samples from Molise from other Central/Southern Italy (Apulia, Calabria and Lazio) samples (LDA_5): Variable weights on the canonical variate.

Variable	CV1
ESA	-0.0498
T2ESE	-0.0636
TERP4	0.3165
TERP2	-0.5711
TERP1	0.1424
1,3DIGL	-0.4316
1,2DIGL	0.5929
Dlnnc	-0.0391
Dlneic	0.1205
SQUA	-0.3378
INS	-0.4113
SAT	-0.1800
LNNC	0.1400
LNEIC	0.1623
SITO	-0.1952

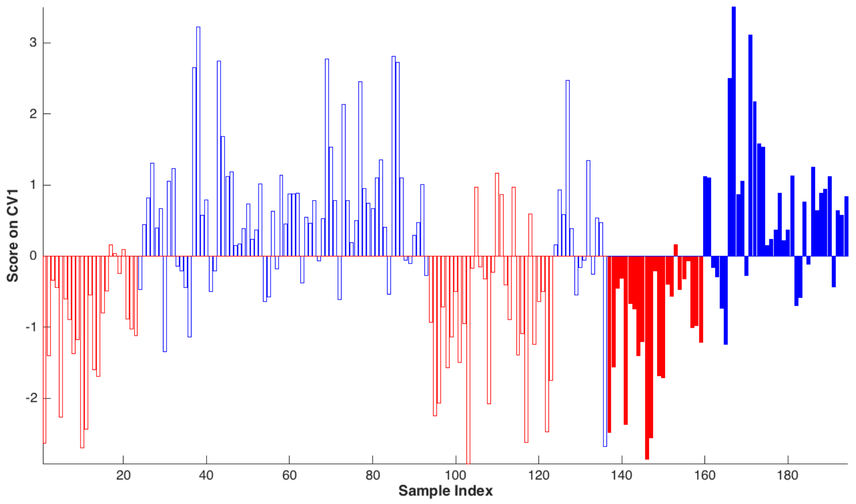

Figure S5. LDA model discriminating oil samples from Apulia from other Central/Southern Italy (Calabria and Lazio) samples (LDA_6): Projection of the training (empty bars) and test samples (filled bars) onto the space spanned by the only Canonical Variates of the LDA model. Legend: Red bars – Apulia; Blue bars – other Central/Southern Italy (Calabria and Lazio).

Table S9: LDA hierarchical model discriminating oil samples from Apulia from other Central/Southern Italy (Calabria and Lazio) samples (LDA_6): Variable weights on the canonical variate.

Variable	CV1
ESA	0.1781
T2ESE	0.1269
TERP4	-0.5657
TERP2	-0.0621
TERP1	0.0348
1,3DIGL	-0.1029
1,2DIGL	-0.5625
Dlnnc	-0.3899
Dlneic	-0.1171
SQUA	1.1012
INS	0.1010
SAT	0.0882
LNNC	0.1889
LNEIC	0.0503
SITO	0.4401

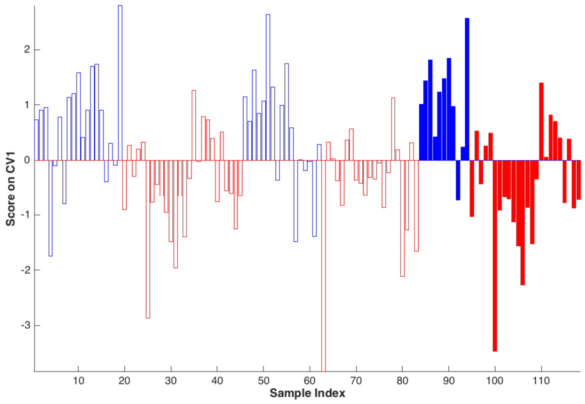

Figure S6. LDA model discriminating between oil samples from Calabria and Lazio (LDA_7): Projection of the training (empty bars) and test samples (filled bars) onto the space spanned by the only Canonical Variates of the LDA model. Legend: Red bars – Calabria; Blue bars – Lazio.

Table S10: LDA hierarchical model discriminating between oil samples from Calabria and Lazio (LDA_7): Variable weights on the canonical variate.

Variable	CV1
ESA	-0.1306
T2ESE	-0.3756
TERP4	0.2548
TERP2	-0.1394
TERP1	0.5501
1,3DIGL	0.1023
1,2DIGL	0.4599
Dlnnc	-0.1319
Dlneic	-0.3074
SQUA	0.5328
INS	0.1883
SAT	0.0197
LNNC	0.5679
LNEIC	-0.2872
SITO	0.1893
